# Graphene and its Derivatives-Based Optical Sensors

**DOI:** 10.3389/fchem.2021.615164

**Published:** 2021-02-05

**Authors:** Xiao-Guang Gao, Ling-Xiao Cheng, Wen-Shuai Jiang, Xiao-Kuan Li, Fei Xing

**Affiliations:** ^1^ College of Biomedical Engineering, Taiyuan University of Technology, Taiyuan, China; ^2^ The Key Laboratory of Weak Light Nonlinear Photonics, Ministry of Education, Nankai University, Tianjin, China; ^3^ The First People’s Hospital of Jinzhong, Jinzhong, China; ^4^ School of Biomedical Engineering, Xinxiang Medical University, Xinxiang, China; ^5^ School of Physics and Optoelectronic Engineering, Shandong University of Technology, Zibo, China

**Keywords:** graphene, fluorescence, surface-enhanced Raman scattering, optical fiber, optical sensors

## Abstract

Being the first successfully prepared two-dimensional material, graphene has attracted extensive attention from researchers due to its excellent properties and extremely wide range of applications. In particular, graphene and its derivatives have displayed several ideal properties, including broadband light absorption, ability to quench fluorescence, excellent biocompatibility, and strong polarization-dependent effects, thus emerging as one of the most popular platforms for optical sensors. Graphene and its derivatives-based optical sensors have numerous advantages, such as high sensitivity, low-cost, fast response time, and small dimensions. In this review, recent developments in graphene and its derivatives-based optical sensors are summarized, covering aspects related to fluorescence, graphene-based substrates for surface-enhanced Raman scattering (SERS), optical fiber biological sensors, and other kinds of graphene-based optical sensors. Various sensing applications, such as single-cell detection, cancer diagnosis, protein, and DNA sensing, are introduced and discussed systematically. Finally, a summary and roadmap of current and future trends are presented in order to provide a prospect for the development of graphene and its derivatives-based optical sensors.

## Introduction

The development of science often starts with unexpected experimental discoveries, which are even inconsistent with previous theories. However, it is through these unexpected discoveries that science has often advanced. For a long time in the past, scientists have believed that at room temperature, free-standing graphene could not exist due to the minimization of its surface energy (Peierls, 1935; Landau, 1937). This speculation was overthrown after the discovery of graphene in 2004 by Andre Geim and Konstantin Novoselov (Novoselov et al., 2004). Since it was first isolated via peeling graphite with adhesives, graphene has attracted tremendous attention from researchers due to its novel properties and wide range of applications (Gilje et al., 2007; Huang et al., 2011; Avouris and Dimitrakopoulos, 2012; Avouris and Xia, 2012; Yang et al., 2013; Gao et al., 2020). As firstly prepared two-dimensional materials, graphene is composed of a one atom-thick planar sheet of *sp* ^2^-bonded carbon atoms perfectly arranged in a honeycomb lattice (Novoselov and Geim, 2007; Nair et al., 2008; Neto et al., 2009). Graphene has many remarkable properties such as high mechanical strength (high Young’s modulus of 1 TPa), thermal properties (high thermal conductivity >3,000 WmK^−1^), and excellent optical characteristics including broadband optical absorption in near infrared and visible range, and nonlinear optical properties, such as saturation absorption (Balandin et al., 2008; Bolotin et al., 2008; Bonaccorso et al., 2010; Koppens et al., 2011; Mak et al., 2012; Han et al., 2014). Due to its unique optical and electrical properties, graphene is widely used in photonic and optoelectronic devices, such as polarizers, modulators, ultrafast lasers, sensors, photodetectors and light-emitting diodes (Fowler et al., 2009; Cheng et al., 2010; Mueller et al., 2010; Sun et al., 2010a; Sun et al., 2010b; Wu et al., 2010; Bao et al., 2011; Kim and Choi, 2012; Liu et al., 2012; Sensale-Rodriguez et al., 2012; Son et al., 2012; Yuan and Shi, 2013; Liu et al., 2014).

**FIGURE 1:**
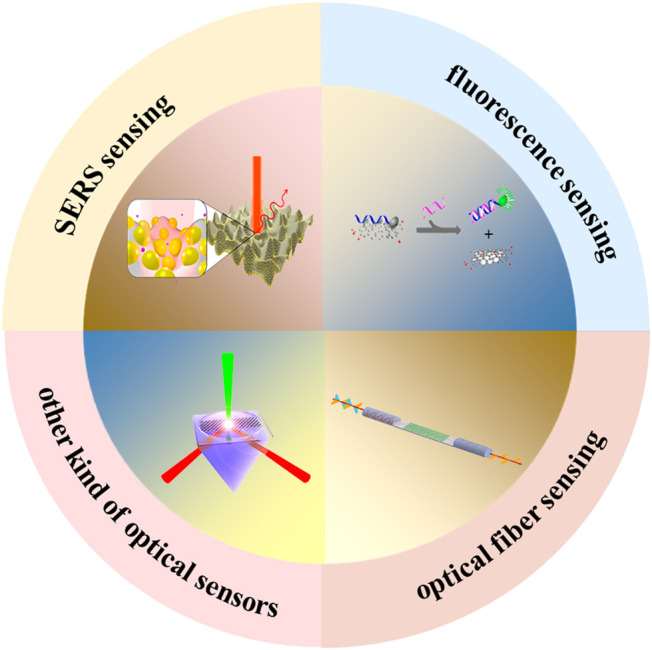
The application of graphene and its derivatives in optical sensors.

By definition, an optical sensor is a device that can convert any external physical stimuli, such as electric field signals, pressure, heat, motion, sound, and biomolecules, into optical or electrical output for reading or further processing. For many years in the past, zero-dimensional material quantum dots, gold nanoparticles, one-dimensional material carbon nanotubes, and nanowires have been widely used in the design of optical sensors (Kong et al., 2001; Modi et al., 2003; Matsui et al., 2005; Frasco and Chaniotakis, 2009; Kuang et al., 2011; Shen et al., 2012). Since then, graphene has been widely employed in the field of optical sensors due to its unique properties. For example, graphene has the following characteristics: the thickness of one-atom layer, large specific surface area, high chemical stability, excellent biocompatibility, and the ability to absorb biomolecules through π-π stacking. These properties allow for the fabrication of gas/chemical vapor, electromechanical, pH, mass, electrochemical, and optical sensors. Graphene-based sensors have shown many advantages over conventional sensors, including reduced dimensions and weight, higher strength, ease of use and fabrication, and reduced cost. Furthermore, graphene derivatives have been also widely used in the field of optical sensors, including graphene oxide (GO), reduced graphene oxide (RGO), and graphene quantum dots (GQDs) (Robinson et al., 2008; Dong et al., 2010; Lipatov et al., 2013; Sun et al., 2013; Ananthanarayanan et al., 2014; Sansone et al., 2014; Wang et al., 2014; Wang et al., 2016). GO is not only an effective fluorescence quencher, but can also be used to achieve the detection of biomolecules with high sensitivity and high selectivity. Graphene prepared through mechanical exfoliation and chemical vapor deposition (CVD) methods has a high quality, but its low yield and high cost have seriously hindered its application. On the other hand, RGO is characterized by high yield and low cost, which render it more suitable for sensing application. In addition, compared with pure graphene, the oxygen functional group contained in RGO makes it easy to interact with biochemical molecules. At the same time, GQDs, another derivative of graphene, have also been extensively used in the field of biochemical sensing GQDs exhibit plenty of advantages such as excellent optoelectronic properties, extremely small size (3–20 nm), stable aqueous colloidal suspensions, ease of functionalization, and tunable fluorescence. Although there have been great achievements regarding graphene and its derivatives-based optical sensors, just a few reviews either highlights graphene-based fluorescence, or only mention graphene-based SERS. To better grasp the whole picture of this area, it is necessary to summarize the recent progress in graphene and its derivatives-based optical sensors. In this review, recent developments in graphene and its derivatives-based optical sensors are summarized, covering aspects related to fluorescence, graphene-based substrates for surface-enhanced Raman scattering (SERS), optical fiber biological sensors, and other kinds of graphene-based optical sensors (Figure 1). Various sensing applications, such as single-cell detection, cancer diagnosis, protein, and DNA sensing, are introduced and discussed systematically. Finally, a summary on recent progress in graphene and its derivatives-based optical sensors is provided, alongside with a proposal for future applications and an outline for researches.

## Fluorescence Sensing

Graphene is composed of a one atom-thick planar sheet of *sp* ^2^-bonded carbon atoms, which exhibits no fluorescence characteristics due to its zero band gap (Novoselov et al., 2012; Zheng and Wu, 2017). The rich oxygen-containing functional groups contained in GO and RGO not only make them to be composed of *sp* ^2^ and *sp* ^3^ carbon atoms, but also give them a band gap and result in fluorescence (Huang et al., 2012; Mathkar et al., 2012). Compared with traditional fluorescent materials, GO has the characteristics of low cost, nontoxic, biocompatible, and environmentally friendly. In addition, GO can achieve adsorption of biomolecules through electrostatic force, hydrogen bonding or π-π interactions, which also provides conditions for the interaction between biomolecules. Therefore, GO has been widely used in fluorescence sensing (Eda and Chhowalla, 2010; Loh et al., 2010; Zhu et al., 2010; Dreyer et al., 2011; Qiang et al., 2014). In this section, the principle and properties of GO fluorescence will be introduced in detail. The application of GO fluorescence in the field of sensing will then be discussed.

**FIGURE 2:**
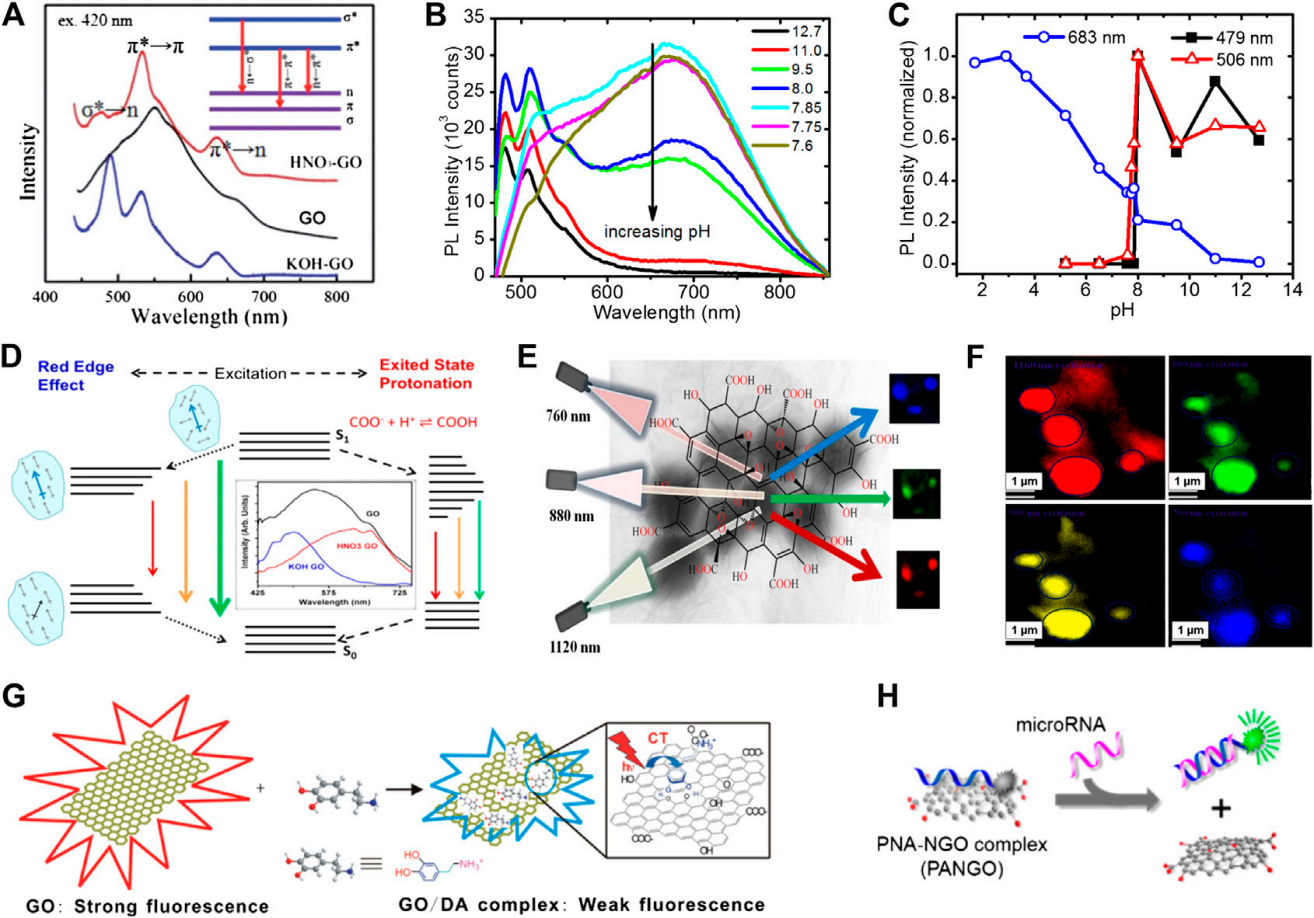
The application of graphene and its derivatives in fluorescence sensing. **(A)** Fluorescence spectra corresponding to GO and GO treated with KOH and HNO_3_; inset: the different electronic transitions (Li et al., 2012). **(B)** The pH-dependent photoluminescence of GO. **(C)** The intensities of photoluminescence at different wavelengths (λ = 683, 506, and 479 nm) and its corresponding pH value (Galande et al., 2011). **(D)** Fluorescence mechanism of GO (Cushing et al., 2014). **(E)** Tunable excitation-wavelength-dependent two-photon imaging. **(F)** Two-photon fluorescence imaging technique based on aptamer-modified GO and its application (Pramanik et al., 2014b). **(G)** High-sensitivity detection of dopamine by using GO as a fluorescence quencher (Chen et al., 2011). **(H)** MicroRNA (miRNA) detection based on GO and peptide nucleic acid (PNA) (Ryoo et al., 2013).

The fluorescence of GO comes from its electronic energy transitions. As displayed in Figure 2A, each fluorescence peak of functionalized GO is derived from the corresponding specific electronic transitions (Zhu et al., 2011). In addition, GO contains various types of oxygen-containing functional groups, such as epoxy (C-O-C), carboxyl groups (COOH), hydroxyl groups (COH), and aromatic rings (C=C), which lead to the overlap of many fluorescence peaks. The position and intensity of the fluorescence peak of GO is highly susceptible to functional groups, solvents, localized domains, and strain. As shown in Figure 2A, the fluorescence peak position and intensity are modified when GO is enriched with OH or COOH groups, respectively (Li et al., 2012). Figures 2B,C show the effect of pH on the fluorescence of GO (Galande et al., 2011). Under acidic conditions, GO exhibits a broad fluorescence peak centered at ∼680 nm. However, as the pH value increases, an amazing phenomenon could be observed. One fluorescence peak (∼680 nm) gradually decreases until it disappears, and another fluorescence peak (∼500 nm) appears under basic conditions. This change in the fluorescence properties caused by PH is mainly due to the excited-state proton transfer, as shown in Figure 2D (Cushing et al., 2014). Under acidic conditions, the COOH group mainly exists in the aqueous solution in the form of ionic COO^−^. Under excitation of incident bean, the two kinds of ions COO^−^ and COOH in the excited-state contributes to the broad fluorescence peak at 668 nm. Furthermore, the fluorescence of GO displays excitation-wavelength-dependent properties due to the giant red-edge effect. As displayed in the Figure 2D, due to the extra relaxation process introduced by the polar solvent, such as water, a giant red-edge effect can be observed When the solvation dynamics occur on a timescale which is orders of magnitude shorter than that of the fluorescence, the solvation is usually completed prior to fluorescence. Therefore, the final fluorescence only undergoes a small redshift.

In fluorescence sensing, when GO is used as a fluorescent chromophore, its fluorescent properties can be modulated by changing its sheet size, chemical composition and other factors. Then, through the process of resonance-energy-transfer and carrier transport, GO is often used as a fluorescence quencher in the field of fluorescence sensing. This dual role of GO as both a fluorophore and a quencher have also brought new ideas to sensor design. Although GO could in principle be used as a fluorescent label, its broad peak limits its sensing performance. Instead, GO is remarkably suitable for near infrared (NIR) biological imaging via two-photon excitation spectroscopy thanks to the giant red-edge effect (So et al., 2000; Tsai et al., 2013; Pramanik et al., 2014b). In 2014, Pramanik et al. developed graphene oxide sheet based two-photon multi-color bio-imaging of multiple drug-resistance bacteria (MDRB), where multicolor imaging is based on the fact that the two-photon fluorescence wavelength of the graphene oxide sheet can be tuned just by varying the excitation energy without changing its chemical composition and size (Galande et al., 2011). As displayed in Figure 2E, the imaging color and luminescence peak position can be tuned from deep blue to red, just by varying the excitation wavelength. Figure 2F displays multicolor two-photon luminescence imaging of methicillin-resistant *Staphylococcus aureus* (MRSA) at different wavelengths excitation. Furthermore, two photon imaging with GO as a fluorescent label has been widely used in the fields of cancer cell imaging and food safety (Pramanik et al., 2014a; Shi et al., 2015; Tchounwou et al., 2015; Wang et al., 2015b). In 2016, Kalluru et al discovered that modified nano-sized GO exhibits wavelength of the excitation dependent fluorescence effect, which is suitable for fluorescence imaging (Kalluru et al., 2016). In addition, the functionalized nano-sized GO can generate a large amount of singlet oxygen through the irradiation of near-infrared light, which is used for photodynamic therapy and photothermal therapy The consequence of experiments also proved that photodynamic and photothermal treatment based on functionalized nano-sized GO can effectively extend the lifespan of mice. In 2019, Song et al. creatively encapsulated nanocrystals doped with rhenium into GO (NCs@GO) (Song et al., 2019). The NCs@GO not only has a strong fluorescence effect in the visible and near infrared bands, but also has satisfactory solubility and biocompatibility. Then the NCs@GO has been utilized not only to achieve high-sensitivity detection of miRNA in cells, but also to realize real-time imaging of tumors.

GO as an effective fluorescence quencher is also widely used in fluorescence sensing. Figure 2G displays the GO being used as an efficient quencher through charge-transfer (Chen et al., 2011). Only when the distance between the donor and the acceptor of the charge is less than 10 Å, the charge transfer process is possible. Such a short distance is often obtained through chemical bonding or physical adsorption. In 2011, Chen et al. a GO-based photoinduced charge transfer (PCT) label-free near-infrared (near-IR) fluorescent biosensor for dopamine. The multiple non-covalent interactions between GO and dopamine can achieve effective fluorescence quenching and high-sensitivity detection of dopamine. In 2013, Wu et al. used GO as a fluorescence quencher to detect heavy metal cations (Li et al., 2013a). The principle involved in the experiment is displays as follows: first, the aptamer must be modified on the surface of GO. When the aptamer captures Hg^2+^, a hairpin structure is formed. In this structure, the charge transfer process between GO and mercury ions causes the fluorescence of GO to be quenched. The present optical sensor shows a limit of detection as low as 0.92 nM and excellent selectivity over various metal ions. The fluorescence sensing based on charge transfer has been applied to the detection of more types of pollutants in environment and agriculture (Zhu et al., 2014; Wang et al., 2015a; He et al., 2015). Fluorescence resonance energy transfer (FRET) is a nonradiative energy-transfer process based on the dipole-dipole interaction (Jung et al., 2010; Kwak et al., 2014). It requires the donor’s emission spectrum to overlap with the acceptor’s absorption spectrum. In the FRET process, GO can serve as an energy donor in which its fluorescence gets quenched by an energy acceptor, such as gold nanoparticles or organic dyes. Furthermore, GO can also act as an energy acceptor, in which it quenches the fluorescence of an energy donor, such as quantum dots and organic dyes. In 2013, Ryoo et al. developed a GO based miRNA sensor, which allows quantitative monitoring of target miRNA expression levels in living cells (Ryoo et al., 2013). The strategy is based on tight binding of GO with peptide nucleic acid (PNA) probes, resulting in fluorescence quenching of the dye that is conjugated to the PNA, and subsequent recovery of the fluorescence upon addition of the target miRNA. The present miRNA sensor allowed the detection of specific target miRNAs with a detection limit as low as ∼1 pM, as well as the simultaneous monitoring of three different miRNAs in a living cell. By inoculating different aptamers and antibodies on the surface of GO, GO-based detection platforms will be used in more fields (He and Cui, 2012; Al-Ogaidi et al., 2014; Iranifam et al., 2016). Compared with traditional organic dye molecules and other types of nanomaterials, graphene and its derivatives have the advantages of photostability and biocompatibility. In addition, graphene and its derivatives could be used not only as fluorophore, but also as fluorescence quenchers in fluorescence sensing, making it widely used in the fields of biosensing and bioimaging. However, the oxygen-containing functional groups in GO form a broad fluorescence peak, which limits its application in biosensing. Therefore, the functional treatment of GO is very critical.

### Graphene-Based Surface-Enhanced Raman Scattering Sensing

Due to the extremely high detection sensitivity and the capability of chemical fingerprints recognition, SERS has been an attractive analytical technique used in various fields (Kneipp et al., 1997; Li et al., 2011; Alvarez-Puebla and Liz-Marzán, 2012; He and Cui, 2012; Zhang et al., 2013). Furthermore, since the phenomenon of graphene based surface enhanced Raman scattering was discovered, the graphene based substrate has attracted wide attention of researchers. Compared with traditional Raman substrates, the introduction of graphene has many advantages, and at the same time it has brought tremendous development. Graphene can not only effectively quench the photoluminescence of fluorescent dyes and drastically eliminate the fluorescence background, but it can also cooperate with typical noble metallic nanoparticles as high-performance SERS substrates. Furthermore, it can serve as an excellent charge transfer-only SERS substrate for understanding the exact role of chemical mechanism without the interference of electromagnetic mechanism (Dalfovo et al., 2014; Lin et al., 2017). In this section, graphene-based SERS substrates and the role played by graphene in graphene-based SERS substrates are summarized. Furthermore, the SERS applications of graphene-based substrates in biomedical areas, including biomolecule detection and bio-imaging are discussed.

**FIGURE 3:**
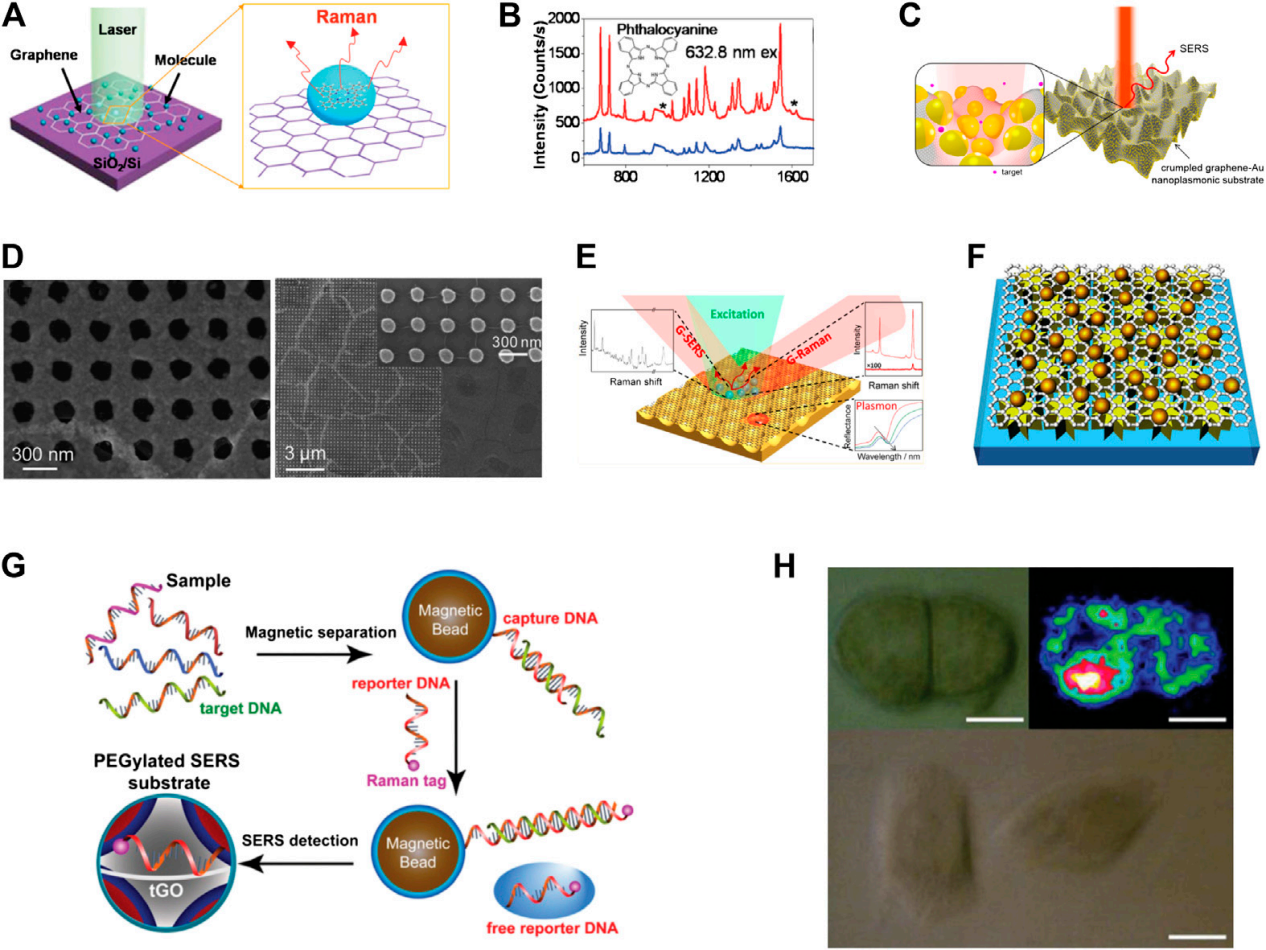
The graphene-based substrates for surface-enhanced Raman scattering (SERS). **(A)** Schematic illustration of the graphene only substrates. **(B)** Raman spectra of Pc molecules on monolayer graphene and on a blank SiO_2_/Si substrate (Ling et al., 2010). **(C)** Schematic of SERS enhancement from a crumpled graphene–Au nanoparticles hybrid structure (Leem et al., 2015). **(D)** Graphene-covered nanoparticles or nanohole arrays for SERS enhancement (Hao et al., 2012). **(E)** Graphene-covered Au nanovoid arrays (Zhu et al., 2013). **(F)** Graphene-separated metal nanostructure substrates (Zhao et al., 2017). **(G)** Schematic illustration of the SERS detection of DNA based on graphene (Duan et al., 2015). **(H)** Optical and Raman images of HeLa 229 cells. The scale bar is 10 µm (Zhang et al., 2015).

Graphene exhibits universal absorption independent of wavelength in the visible range, and has plasmon resonance in the terahertz. The Raman enhancement effect of graphene is mainly attributed to the charge transfer between graphene and various molecules, which result in a chemical enhancement. In 2010, Ling et al. explored the possibility that graphene could be used as a substrate for enhancing Raman signals of adsorbed molecules (Lai et al., 2018). Several different types of molecules, used as Raman probe, were deposited through an identical process both on graphene and on a SiO_2_/Si substrate using vacuum evaporation or solution soaking. By comparing the Raman signals of molecules on graphene and on a SiO_2_/Si substrate, they demonstrated that the intensities of the Raman signals on graphene were much stronger than on a SiO_2_/Si substrate. Figure 3A displays the graphene-only substrates. In Figure 3B, the intensities of the Raman signals of phthalocyanine (Pc) on graphene are much stronger indicating that graphene can enhance the Raman signals of these molecules.

In addition, graphene derivatives-based substrates have also been extensively studied. In 2013, Liu et al. explored the effects of pH values on SERS intensities of some aromatic molecules on GO. They concluded that the GO-mediated SERS is associated with charge-type selectivity according to electrostatic interactions (Liu et al., 2013). In 2016, Yin et al. explored the SERS effects of RGO with different degrees of reduction (Yin et al., 2016). They found that the Raman intensities of RGO decreased with increasing the reduction duration from 2 to 60 min, while the strongest Raman intensity of R6G molecules was observed for 10 min. Graphene-only substrates provide a better way to understand the chemical mechanism of SERS, but their enhancement effect remains still no strong enough for application. Therefore, it is necessary to develop graphene–metal substrates for highly sensitive SERS analysis. Figure 3C displays the graphene-supported nanostructures substrate. Leem et al. developed a mechanical self-assembly strategy to enable a new class of 3D crumpled graphene-gold (Au) nanoparticles hybrid nanoplasmonic structures for SERS applications (Leem et al., 2015). The 3D crumpled graphene-Au NPs exhibit at least one order of magnitude higher SERS detection sensitivity than that of conventional, flat graphene-Au NPs. The hybrid structure is further adapted to arbitrary curvilinear structures for advanced, *in situ*, nonconventional, nanoplasmonic sensing applications. The graphene-covered metal nanostructures substrates are displayed in Figures 3D,E (Hao et al., 2012; Zhu et al., 2013). In this structure, graphene can serve not only as a protective layer, so that the metal microstructure is not oxidized and corroded, but also to avoid direct contact between the biomolecules and the metal microstructure, thus allowing to exclude the side photocatalytic reaction. The graphene-separated metal nanostructure substrate is displayed in Figure 3F. Zhao et al. developed an efficient SERS substrate by sandwiching graphene between Au NPs and electron beam lithography-fabricated Ag nanostar arrays (NSAs). The fabricated hybrid structure exhibits 137-fold enhancement of the Raman response of graphene, with a limit of detection of 0.1 pM for rhodamine 6G molecules (Zhao et al., 2017).

Undoubtedly, graphene has been widely employed in the design of SERS substrates. Graphene has overcome some of the limitations of the SERS substrates since it endows the SERS substrates with better stability, sensitivity, reproducibility, and biocompatibility. More importantly, high-performance graphene based substrates have great application potential in numerous fields. Various biomarkers and biomolecules, such as DNA, nucleosides, proteins, bacteria, and fungi have been successfully detected through the employment of multiple graphene-based substrates (Kang et al., 2010; Li et al., 2013b; Lin et al., 2014; Duan et al., 2015; Xu et al., 2015; Ouyang et al., 2017). In addition, graphene-based substrates have been shown to be effective for bio-imaging, cancer diagnostics, drug delivery, photothermal therapy and chemotherapy (Ilkhani et al., 2016; Cialla-May et al., 2017). Figure 3G displays an example of DNA detection using graphene-based SERS sensing. In 2015, Duan et al. developed a new type of SERS substrate with thiolated graphene oxide (tGO) nanosheets sandwiched between two layers of closely packed plasmonic nanoparticles (Duan et al., 2015). Herein, tGO can play multifunctional roles as a 2D scaffold to immobilize interfacially assembled plasmonic nanoparticles, a nanospacer to create SERS-active nanogaps between two layers of nanoparticle arrays, and a molecule harvester to enrich molecules of interest via π–π interaction. Furthermore, they demonstrated that an SERS assay based on the PEGylated substrate, in combination with magnetic separation, allows for sensitive, multiplexed “signal-off” detection of DNA sequences of bacterial pathogens. Cell imaging is a powerful method to reveal mechanisms and cellular processes and to diagnose diseases. In Figure 3H, Zhang et al. developed the Raman-fluorescence dual imaging of cells based on a multifunctional GO/AuNPs/2-aminoethanethiol (AET)/fluorescein isothiocyanate hybrid platform (Zhang et al., 2015). Raman imaging of HeLa 229 cells with GO/AuNPs/p-ATP hybrids showed excellent performance as the characteristic peaks of p-ATP at 1,078, 1,137, 1,330, 1,435, and 1,590 cm^−1^ could be clearly observed. In 2017, Zhang et al. prepared Au triangular nanoarray/graphene/Au nanoparticles sandwich structure as the SERS substrate (Zhang et al., 2017). They have proved that the high-temperature annealing process can effectively reduce the distance between the Au nanoparticles and the Au triangular nanoarray, and achieve effective amplification of the Raman signal. Then the excellent SERS platform has been used for high sensitivity detection of mercury ions (8.3*10^−9^ M). In 2018, Zeng et al. successfully synthesized silver nanospheres coated with graphene oxide (Ag@NGO) (Zeng et al., 2018). The hybrid nanomaterials not only can effectively enhance the Raman signal, but also its stability is greatly improved due to the presence of GO. Then they proved that Ag@NGO nanoparticles can not only be used as an effective nano-probe to monitor intracellular biological molecular, but also can serve as a drug delivery nano-carriers as well by π-π interaction with anticancer drug DOX. In 2020, Choi et al. synthesized GO-coated gold nanoarrays in the shape of tooth to achieve effective Raman signal enhancement (Choi et al., 2020). Then the GO hybrid structure were utilized to achieve rapid and highly sensitive detection of dopamine (10^−4^ to 10^−9^ M). In additional, SERS based on a reliable graphene-based substrate has been successfully used to discriminate various environment pollutants, including organic pollutants, heavy metal ions, pathogens, and antibiotics. Compared with traditional SERS substrates, graphene has proper modification and improved biocompatibility, which also makes graphene-based SERS substrates widely used in biosensing, drug delivery, and bioimaging fields. However, graphene-based substrates also need to address many difficulties and challenges. First of all, the stability of graphene-based substrates needs to be improved, especially when various biochemical reactions occur on the surface of graphene. In addition, non-specific adsorption on the graphene surface will also cause serious disturbances. Therefore, it is urgent to develop graphene based substrates with high stability and high specificity.

### Graphene-Based Optical Fiber Sensing

Optical fiber sensors have received world-wide attention due to their high sensitivity, small size, good anti-electromagnetism disturbance ability and other potential advantages. As the first prepared two-dimensional material, graphene has been used extensively in the design of optical fiber sensors due to its unique optical properties (Yao et al., 2014a; Zhang et al., 2014; Dash and Jha, 2015; Li et al., 2015; Zeng et al., 2015). In this section, the principles of different types of graphene-based optical fiber sensors and their applications in biochemical sensing are introduced in detail. It was shown that graphene had a great potential in the optical fiber sensing technology.

**FIGURE 4:**
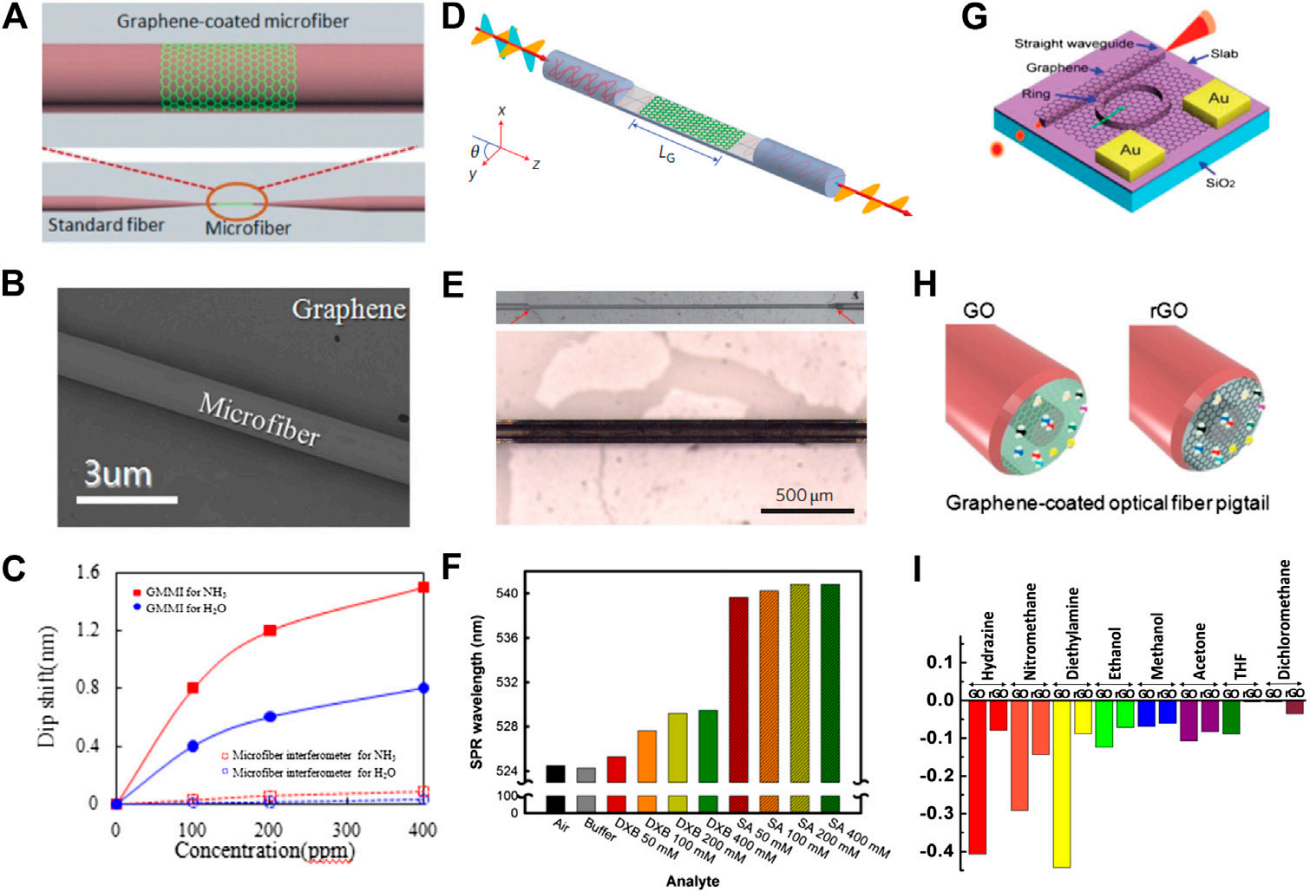
The graphene based fiber optical sensors. **(A)** Schematic diagram of the graphene/microfiber hybrid waveguide (Shivananju et al., 2017). **(B)** Scanning electron microscopy (SEM) image of the graphene-coated microfiber waveguide (Yao et al., 2014a). **(C)** Graphene-coated microfiber waveguide used for high-sensitivity gas sensing (Yao et al., 2014b). **(D)** Schematic model of the fiber-to-graphene coupler based on a side-polished optical fibre. **(E)** Optical images of a laterally polished optical fiber and of a planar section of the optical fiber covered by few-layer graphene (Bao et al., 2011). **(F)** The fiber-to-graphene coupler were used for high-sensitivity protein sensing (Kim et al., 2013). **(G)** Illustration of graphene-coated optical microring resonator (Gan et al., 2015). **(H)** Schematic of the end of an optical fiber pigtail with a graphene coating on pinhole (Shivananju et al., 2017). **(I)** Comparative plots of the sensing responses of GO and RGO-coated polymer optical fibers (Some et al., 2013).

Graphene based fiber optical sensors with various device configurations for biochemical sensing are displayed in Figure 4. Figure 4A shows a schematic structure of a thin layer of graphene wrapped around a sub-wavelength-diameter (1 μm) tapered single-mode microfiber (Shivananju et al., 2017). In this structure, tapered or micro fibers are usually prepared via a chemical etching process or a gently stretching process while heating over a flame or using a heated filament. During the preparation process, the fiber core becomes thinner, causing the evanescent wave to reach the outside and thus being exposed to the surrounding medium through the graphene. A surface plasmon resonance (SPR) sensor is obtained by placing a graphene layer over the tapered region, and is widely used in gas sensing. In 2014, Yao et al. developed an all-optical NH_3_ gas sensor based on graphene/microfiber hybrid waveguide (GMHW) (Yao et al., 2014a). During the fabrication process of the GMHW sensor, they transferred the graphene prepared via CVD to the MgF_2_ substrate. The graphene/MgF_2_ substrate was then fixed on a translation stage and the microfiber was attached onto the graphene. The SEM image of the graphene-coated tapered microfiber is depicted in Figure 4B. The adsorption of NH_3_ can change the effective refractive index of the GMHW. The wavelength shift induced by the NH_3_ absorption is spectrally demodulated using a microfiber-based Mach–Zehnder interferometer. The GMHW has been demonstrated to have a high sensitivity of ∼6 pm/ppm, and a resolution of 0.3 ppm. In 2014, Yao et al. observed the enhancement of the surface evanescent field by graphene cylindrical cladding (Yao et al., 2014b). It was found that the light in the fiber core can be effectively modulated by graphene, improving the detection sensitivity of the hybrid waveguide. The experimental results for gas sensing verified the theoretical prediction, and ultra-high sensitivities of ∼0.1 ppm for NH_3_ gas detection and ∼0.2 ppm for H_2_O vapor detection were achieved (Figure 4C).

Figure 4D displays a schematic diagram of a fiber-to-graphene coupler based on a side-polished or D-shaped optical fiber (Bao et al., 2011). The optical images of a laterally polished optical fiber and of a planar section of optical fiber covered by few-layer graphene are displayed in Figure 4E. The working principle of this structure is based on the interaction of the evanescent wave with the surrounding environment. In 2014, Kim et al. developed graphene-based D-shaped fiber optic SPR for biochemical sensing applications (Kim et al., 2013). A graphene film synthesized via thermal chemical vapor deposition is transferred onto the sensing area of the optical fiber. The detection mechanism of this sensor is based on the principle that the SPR signal changes according to the refractive indices of the analytes. In this experiment, the LED light (524 nm) is polarized and coupled into the graphene-coated fiber sensor. The change in the polarized light due to the change in the refractive index, caused by biomolecule interaction, is captured by spectrometer. Figure 4F displays the bar chart for SPR wavelengths for each analyte. The SPR peaks appear at ∼ 524 nm for the buffer case and the case without analyte, 525.3–529.5 nm for biotinylated double crossover DNA (DXB) samples, and 539.6–540.8 nm for protein streptavidin (SA) samples. The consequences of this experiment verify the sensitivity and selectivity of graphene-coated fiber sensors for protein and DNA detection. Figure 4G displays a schematic diagram of the optical ring resonator covered with graphene (Gan et al., 2015). The graphene-coated optical ring resonator sensor relies on light-analyte interaction to convert the presence of biochemical molecules into quantitatively measurable optical signals. The resonant wavelength of light circulates along the graphene-coated ring resonator and has an evanescent field that reaches several hundred nanometers into the biochemical molecules to interact repeatedly with the analytes near the resonator surface. Graphene-coated optical ring resonators are more sensitive and can achieve high quality factors. The schematic diagram of the graphene film coated on the optical fiber end pigtail is displayed in Figure 4H. In this approach, light is needed for single-ended probes with a common input and output path and works in reflection mode. In 2013, Some et al. developed novel one-headed polymer optical fiber sensor arrays using hydrophilic GO and hydrophobic RGO (Some et al., 2013). The working principle of this optical gas sensor is based on the change in the reflection of light by the interfacial layer at the fiber end facet, which in turn is induced by changes in the refractive index of the GO and RGO layers. The response of the GO- and RGO-coated sensors for volatile organic compounds, including hydrazine, nitromethane, diethylamine, ethanol, methanol, acetone, tetrahydrofuran (THF), and dichloromethane (MC), are displayed in Figure 4I. Besides, the eco-friendly physical properties of GO allow for faster sensing and higher sensitivity when compared to RGO even under extreme environments of over 90% humidity, making it the best choice for gas sensor. Although graphene-based optical fiber sensors have been widely used, there are still many difficulties to overcome in reality. It is difficult for graphene to be perfectly transferred to the surface of the optical fiber, and its thickness and quality are also difficult to control. Then, the stability and repeatability of graphene-based optical fiber sensors still need to be improved.

### Other Kind of Graphene-Based Optical Sensors

The interaction between incident beam and graphene mainly includes interband and intraband transitions. In the far infrared and THz bands, the electronic response consists mainly of intraband transition (free carrier response), which can be well described by the Drude model. Instead, in the near infrared and visible range, the absorption of graphene consists mainly of interband transition, which is wavelength independent. Since the absorption of single layer graphene is only 2.3%, a variety of structures have been designed to enhance the absorption of graphene (Mueller et al., 2010; Koppens et al., 2011; Wang et al., 2013b; Pirruccio et al., 2013; Pospischil et al., 2013).

In 2013, Ye et al. discovered that graphene exhibits strong polarization dependent optical absorption under total internal reflection (Ye et al., 2013). Compared with the limited universal absorbance of 2.3%, a larger absorption was observed in monolayer, bilayer, and few-layer graphenes for transverse electric (TE) wave under total internal reflection. Based on the polarization-sensitive absorption effect, Wang et al. proposed a method to accurately count the number of layers for both exfoliated and chemical vapor deposition graphene on transparent substrate (Wang et al., 2013a). Their method is useful for graphene tests on transparent substrates, which is different from the commonly used SiO_2_/Si substrate.

**FIGURE 5:**
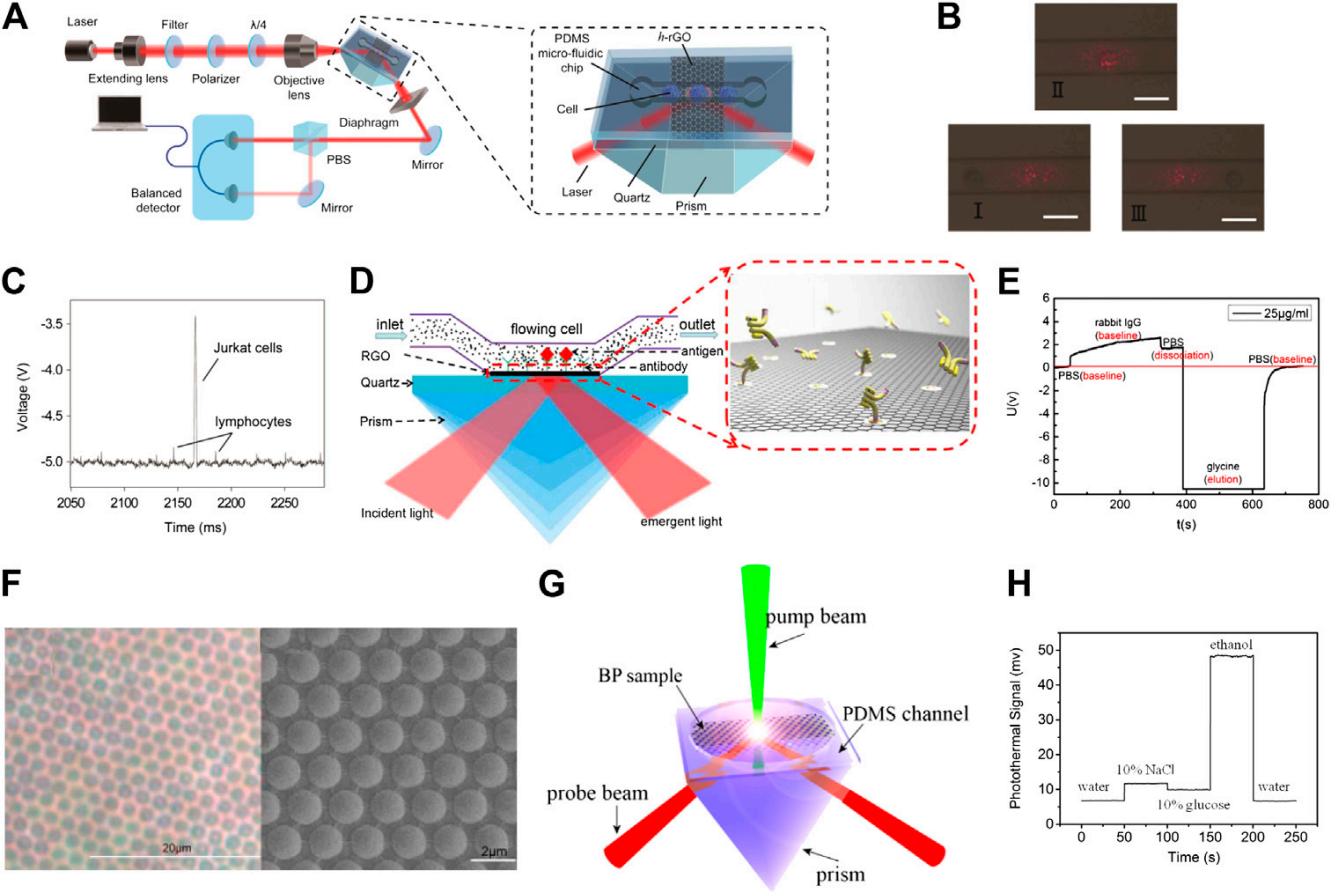
**(A)** Ultrasensitive sensing of single cell using graphene-based optical sensor (Xing et al., 2014). **(B)** Optical images of the RGO detection window as lymphocytes roll across it. The scale bar is 15 μm. **(C)** Discrete time-dependent changes in signal that correspond to mixed lymphocytes and Jurkat cells as they roll across the detection window. **(D)** The RGO-based optical sensor for detecting specific protein (Jiang et al., 2017b). **(E)** Signal changes caused by the interaction of antigen and antibody. **(F)** Optical microscopy and SEM image of reduced graphene oxide microshell (RGOM) (Jiang et al., 2017a). **(G)** Schematic diagram of the photothermal detection experimental setup (Gao et al., 2018b). **(H)** Time-dependent changes in photothermal signal when different kinds of liquid medium are injected.

In addition, the polarized absorption optical properties of graphene have also been used in sensor design. In 2014, Xing et al. used the polarization dependent optical absorption of graphene in combination with microfluidic technology to achieve high sensitivity detection of cancer cells (Xing et al., 2014). They obtained a graphene-based optical refractive index sensor with high resolution (1.7 × 10^−8^) and sensitivity (4.3 × 10^7^ mV/RIU), as well as an extensive dynamic range. Figure 5A displays a schematic diagram of the graphene-based optical sensor. The probe beam is generated by a He-Ne laser with a wavelength of 632.8 nm, and its direction of polarization is modulated by a polarizer and quarter-wave plate. The probe beam is then focused onto the center of its microfluidic channel. The inset of Figure 5A displays the schematic of the graphene-based optical single-cell sensor (GSOCS), which consists of a polydimethylsiloxane (PDMS) microfluidic chip/h-RGO/quartz sandwich structure on the prism. After interacting with graphene, the probe beam is separated into *s*- and *p*-polarized beams by a polarization beam splitter. The difference in intensity of the two modes is measured by a balanced detector. The researchers regarded cancer cells and normal cells as microspheres with different refractive indices. When cancer cells and normal cells pass through the surface of graphene, the refractive index changes induced are different, thus enabling the detection of cancer cells. Figure 5B illustrates the passing of cells through the microfluidic channel. In Figure 5C, the high and low voltage levels represent the signals from the cancer cell and normal cells. The GSOCS can achieve high sensitivity detection of cancer cells, which has significance for the early diagnosis and treatment of cancer. In 2017, Jiang et al. used graphene-based optical sensors to achieve high sensitivity detection of rabbit IgG (Jiang et al., 2017b). Figure 5D displays the schematic of the graphene-based optical sensor. In this process, graphene acts not only as a molecular link layer to inoculate antibodies, but also as a sensing layer to detect changes in the refractive index caused by the interaction of antigens and antibodies. Figure 5E displays a real-time measuring result of the biosensor after fabrication and biochemical treatment. The whole process of biomolecular interaction between antigen and antibody can be observed clearly, as shown in Figure 5D, which is similar to the dynamic process of biomolecular interaction of SPR-based sensor. Compared with a commercial SPR apparatus, graphene-based optical sensors can achieve higher sensitivity detection. The graphene based optical sensor shows a satisfactory response to rabbit IgG with a minimum concentration of 0.0625 μg/ml. Furthermore, Jiang et al. developed a reduced graphene oxide microshell (RGOM)-based optical biosensor for the determination of goat anti-rabbit IgG (Jiang et al., 2017a). In Figure 5F, the RGOM was prepared through a self-assembly of monolayer of monodisperse polystyrene microspheres. Through high temperature reduction, the RGOM was fabricated to inoculate rabbit IgG. Compared with RGO, the periodic microshells allowed for a simpler functionalization and modification of RGOM with biomolecules. This method is promising for immobilizing biomolecules on graphene surfaces and for the fabrication of biosensors with enhanced sensitivity.

In 2018, Gao et al. used the polarization dependent absorption of two-dimensional materials and a modulated pump beam to measure their photothermal signal (Gao et al., 2018a; Gao et al., 2018b; Gao et al., 2018c). Photothermal detection (PTD) is a refractive index sensing optical technique, in which a modulated pump beam is absorbed by two-dimensional materials, leading to a local change in the refractive index of the photothermal medium. The propagation of the probe beam at different wavelengths is modified by the produced periodical change of the refractive index. Figure 5G displays the schematic diagram of the PTD experimental setup. The pump beam used in the experiments is produced by a semiconductor laser at a wavelength of 532 nm. The pump laser is linearly polarized, with its polarization angle being changed via a half-wave plate, and is regulated to a certain frequency through an acousto-optic modulator. After reflecting on the mirror, the pump beam is then focused onto the samples through the objective lens. The probe beam is generated by a He–Ne laser at 632.8 nm and its polarization is modulated by the polarizer and a one-half plate. After interacting with the sample, the probe beam is split into *s*- and *p*-polarized beams by a polarization beam splitter. The detector measures the difference between *s*- and *p*-polarization caused by the modulated pump beam. By exploring the effect of the polarization angle of the pump beam on photothermal signal, Gao et al. explored the photothermal anisotropy of BP and ReSe_2_. Furthermore, the photothermal anisotropies of BP/ReSe_2_ heterostructures were also explored. The photothermal contrasts of samples were observed to change with different stacking angles indicating that the photothermal anisotropy of heterostructures is dependent on the stacking angle. These findings provide new prospects for designing novel optical devices based on two-dimensional anisotropic materials. In addition, the PTD technique has been used for identification of the crystalline orientation of anisotropic two-dimensional materials on a transparent substrate. Compared with traditional crystal orientation determination methods, the PTD overcomes typical challenges associated with transparent substrates, including insulating and rough surfaces, and enables the unambiguous identification of crystalline orientation. Figure 5H displays the effect of different types of liquid media on the photothermal signal of graphene. Gao et al. proved that the thermal conductivity, heat capacity, and thermally-induced refractive index changes of the liquid medium will cause the change in the photothermal signal, indicating that the PTD technique can be implemented into a new type of optical sensor.

## Conclusion and Outlook

In summary, the recent progress in graphene and its derivatives-based optical sensors have been reviewed, covering aspects related to fluorescence sensing, graphene-based substrates for surface-enhanced Raman scattering, optical fiber biological sensors, and other kinds of graphene based optical sensors. The last few years have witnessed a dramatic increase in research effort on graphene-based optical sensors, both at the fundamental level and from a technological point of view. Graphene and its derivatives-based optical sensors have several advantages, including fast response, high sensitivity, and high flexibility, and are widely used in cancer cell imaging, DNA sensing and protein detection. Although tremendous efforts have been devoted to this research field over the past several years, there still undeniably remain several significant issues that are required to be addressed and explored. The challenges involved include not only the strategies to synthesize graphene and its derivatives but also the integration of sensors into practical applications. In the synthesis process, a major challenge is to synthesize high-quality graphene, and its properties can be tuned through effective chemical methods. Another challenge is to achieve a low-cost, environmentally friendly preparation method for graphene and its derivatives. For the practical application of optical sensors based on graphene and its derivatives, the primary issue is to develop high-sensitivity and high-specificity sensors, while another important objective is to enable miniaturization for integration into wearable devices. Overcoming the above challenges will not only boost graphene and its derivatives toward applications in optical sensing technology, but will also greatly advantage peoples’ life.

## Author Contributions

X-GG and L-XC conceived the idea and designed the manuscript. X-GG wrote the paper. W-SJ, X-KL, and FX participated in the collection and sorting of relevant materials. All authors discussed the results and commented on the manuscript.

## Funding

This work was supported by the Natural Science Foundation ofChina (Grant 62005189), Natural Science Foundation of Shanxi Province (201901D211071), the open foundation of Key Laboratory of Weak Light Nonlinear Photonics, Ministry of Education (OS20-2), the Science and Technology Research Project of Henan Province (182102311130), the Key Scientific Research Items of Henan Province Colleges and Universities (20A416006), the Doctoral Research Grant Program of Xinxiang Medical University (XYBSKYZZ201719), the Natural Science Foundation of Henan Province (Grant No. 182300410107).

## Conflict of Interest

The authors declare that the research was conducted in the absence of any commercial or financial relationships that could be construed as a potential conflict of interest.
